# Supplementary material for: Identification of a necroptosis-related gene signature for making clinical predictions of the survival of patients with lung adenocarcinoma
Source: PeerJ. 2024 Jan 8;12:e16616. doi: 10.7717/peerj.16616 (PMC10782958; doi:10.7717/peerj.16616)
Supplement: Supplemental Information 3 [file peerj-12-16616-s003.docx]

| Table S1 Necroptosis-related gene list | | | | | |
| --- | --- | --- | --- | --- | --- |
| RIPK1 | MIR21 | CTSS | RIPK2 | KRT2 | RPS4X |
| MLKL | TNFRSF10B | TNFRSF25 | UBE2D3 | KRT9 | RPL34 |
| RIPK3 | AURKA | DSTYK | TPM2 | NCL | SCYL2 |
| ZBP1 | MYH9 | RNF31 | UGDH | ELAVL1 | THRAP3 |
| CASP8 | CASP10 | IKBKB | TUFM | IGF2BP3 | TRIP6 |
| TNF | PLK1 | CHUK | TUBB4A | AP2S1 | TMOD3 |
| CYLD | SQSTM1 | BUB1B | TRAF6 | PAWR | SNRPF |
| ITPK1 | TNFRSF10A | IRAK1 | TET2 | RPL26 | SRSF6 |
| IPMK | SOAT1 | MAPK3 | COPB2 | RPL9 | SRSF4 |
| MAP3K7 | HGF | TAB2 | ARHGEF2 | RPL4 | SRSF3 |
| TRPM7 | SRC | EIF2AK3 | ATF4 | RPL7 | CLINT1 |
| FADD | ANXA1 | NQO1 | EIF4EBP1 | PSMC4 | CEP170 |
| PELI1 | FPR1 | HSPA8 | CLTC | RPS13 | AMBRA1 |
| PGLYRP1 | CASP2 | IKBKG | CALM2 | RPS23 | CPSF6 |
| SPATA2 | MIF | BDNF | LTBP1 | RPS26 | COPS2 |
| CASP6 | KL | BIRC3 | DDX5 | RPS29 | CARD6 |
| TP53 | TNFRSF21 | XBP1 | PPP1CB | RPL10A | CAPN7 |
| TNFRSF1A | BCL2L1 | BECN1 | PRSS1 | RPS12 | ATXN2L |
| TNFAIP3 | BMI1 | TICAM1 | TUBA4A | RPL12 | ADRM1 |
| UCHL1 | BBC3 | SOX17 | KRT19 | SVIL | LRRFIP2 |
| SIRT3 | AVEN | HSPA4 | KRT6A | SLC25A6 | PREB |
| TNIP1 | ZNF7 | TAB1 | LGALS3 | SNRPE | PKP3 |
| STING1 | PTEN | BNIP3 | FUS | CAPZA1 | ZNF24 |
| SERTAD1 | ESR2 | RBCK1 | EMD | EIF4B | ZFP36 |
| TRAF2 | GSK3A | UBC | HSPA1A | CUL4A | YBX1 |
| CFLAR | C5 | TAB3 | GBE1 | CNBP | ZNF217 |
| MAPK14 | SP1 | AGFG1 | MYO5A | BANF1 | ZC3HAV1 |
| HMGB1 | EGR1 | EIF2A | PARK7 | AP2B1 | GCC2 |
| FAS | C7 | TNIP2 | RPS10 | MAP1LC3A | FAF2 |
| BIRC2 | C9 | MIB2 | RPL35 | RNGTT | HELQ |
| AIFM1 | C6 | H1-5 | S100A10 | RPL3 | GTF3C3 |
| KLHDC10 | ATG5 | DIRAS3 | S100A4 | PCBP1 | CDC42BPG |
| GSK3B | CD74 | C20orf204 | CALM1 | PRPF8 | RPL17 |
| XIAP | NOX4 | JAK1 | CCT5 | PYCARD | RPS25 |
| MYC | TNFSF12 | EGFR | AFG3L2 | SLC25A10 | RPS27L |
| TNIP3 | MKRN1 | FLT3 | AHSG | TUBB6 | TNRC6B |
| GJB1 | RCN1 | IDH2 | MAP1B | IGF2BP1 | TWF1 |
| DNM1L | UHRF1 | IDH1 | PPM1B | HNRNPF | TANC2 |
| PTGES3 | AIFM2 | HMOX1 | PKM | HSPA1B | ALYREF |
| BRD4 | SGK1 | MMP13 | PABPN1 | IQSEC1 | CPSF2 |
| RB1 | PAK1 | NPM1 | PKP2 | IL24 | CPSF3 |
| SIRT2 | CDC7 | G6PD | ULK1 | MPP1 | DCD |
| DAPK1 | CERK | GJA1 | XRCC6 | MYL6 | MGA |
| FKBP1A | CTSB | TSC2 | XRCC5 | IVNS1ABP | RAI14 |
| MIR425 | CD40LG | VIM | UBE2L3 | MVP | RPL38 |
| EZH2 | OGT | CASP1 | NDUFA4 | PDIA4 | OTUD4 |
| NFE2L2 | BID | CAD | NRIP1 | PDLIM7 | PLEKHA5 |
| CD274 | IFNB1 | DRD2 | NSUN2 | RPLP2 | TXNIP |
| CHMP4B | ID1 | ATP2A1 | OPTN | RPS16 | WRNIP1 |
| CXCL5 | MIR155 | KRT18 | RPS20 | RPLP1 | ZNF146 |
| MIR29B1 | TLR3 | RPS19 | STK38 | TPM4 | LIMCH1 |
| BCL2 | KIAA1191 | TNNT2 | SFPQ | SRSF9 | LACTB |
| AXL | TRADD | TPM1 | TUBB4B | SRSF1 | G3BP2 |
| MERTK | TP63 | EPAS1 | SLC25A5 | CCNT1 | FAM83D |
| TYRO3 | IL1A | APC | LITAF | DYNLL1 | HEMGN |
| SIRT6 | PADI4 | AHR | RPL15 | DNAJA1 | HSPBAP1 |
| NAT2 | MIR22 | ACTB | KRT10 | DDX17 | GOLGA3 |
| SLC39A7 | MIR221 | LRP1 | LGALS1 | CCT3 | HRNR |
| USP22 | MIR214 | VCP | KLF6 | BCLAF1 | GLTP |
| SFTPA1 | MIR101-1 | YWHAE | KRT16 | AGO2 | NUDT21 |
| PANX1 | MIR485 | YWHAG | KRT7 | RPL23A | SERBP1 |
| FLOT1 | MIR101-2 | LEF1 | MYO1C | KRT86 | SEC16A |
| FLOT2 | CTSD | NOD2 | EEF1A1 | NME8 | SRRM2 |
| PDCD6IP | CLEC7A | PIKFYVE | HNRNPU | HNRNPH1 | SRP14 |
| DIABLO | OTULIN | EEF2 | FLII | HNRNPL | SRSF10 |
| FASN | STAT3 | BAX | ACTC1 | HOOK1 | ESYT2 |
| CDK9 | HPRT1 | MAPKAPK2 | KHDRBS1 | HNRNPH3 | ERH |
| TIMM50 | FAP | PRDX1 | PNKD | FIP1L1 | MLF2 |
| SLC25A37 | TRIM24 | KIF11 | PSMA3 | PDIA6 | RBM14 |
| PPP1R3G | CHL1 | SLC16A1 | RPL7A | RPL6 | PRPF40A |
| MIR7-1 | PGAM5 | KRT8 | RPS14 | RIOK1 | POF1B |
| NFKBIA | TGFBR1 | KRT5 | RPS17 | RPL23 | VBP1 |
| AURKC | CDKN2A | KRT1 | RPS24 | RPL28 | YTHDC1 |
| NGFR | ACVR1B | KRT14 | RPS27 | RPL29 | AKAP8L |
| FMR1 | PYGM | NLRP3 | RPS3 | SSBP1 | AKNA |
| GNLY | CCL2 | HIF1A | RPS6 | SF1 | TRAFD1 |
| PARP1 | MEFV | HSP90AB1 | RPL13A | TRA2B | CALML5 |
| HTRA2 | DPEP1 | HSPD1 | RPL13 | TRA2A | PNN |
| HSPA5 | AIM2 | HSPA9 | TANK | SYNCRIP | ELP1 |
| PRKAA2 | UBR2 | PINK1 | TCOF1 | SRSF7 | KCTD5 |
| PRKAA1 | CHMP1A | RPL5 | TNFRSF8 | CCT8 | RBM25 |
| PITPNA | GPX4 | TNFRSF1B | TRIM28 | ADAMTSL4 | UBL4A |
| TRAF5 | LAMP2 | TPM3 | TUBA1C | CCT6A | APOOL |
| METTL3 | MPRIP | TRAF3 | S100A6 | DNAJA2 | CRTAM |
| FNDC4 | MTOR | SLC25A13 | TCP1 | BAG2 | CTAG2 |
| FNDC5 | BRAF | BAP1 | SLC30A9 | ATAD3A | LRRC59 |
| FASLG | AFP | DDX3X | CALM3 | RCC2 | PPP1R12C |
| TXN | ACHE | RANBP2 | ASIC1 | RFWD3 | PALMD |
| RALBP1 | NFAT5 | RPL11 | CADM1 | WDR77 | TLE6 |
| TP53I3 | VIL1 | PRPS1 | CALU | LARP1 | ZSCAN20 |
| PRKN | CXCL1 | SLC25A1 | RBMX | MYL12A | ZKSCAN4 |
| GSDMD | SHARPIN | MYO6 | RPL22 | MYO1D | H1-2 |
| HSP90AA1 | MAPK1 | FLNC | RPL27 | MYO1B | RPL39 |
| STUB1 | CBL | HNRNPA2B1 | PCM1 | MYCBP | SP6 |
| TNFSF10 | GSN | HNRNPA1 | PABPC1 | HNRNPM | CHTOP |
| CDC37 | TBK1 | IKBKE | COPA | MYL6B | UBAP2 |
| NFKB1 | MAPK8 | MYH14 | PLEC | GOSR1 | ZAN |
| RELA | RARG | IL4 | TUBA1B | MRPS12 | GSDME |
| TRPC6 | CTSH | NUP214 | XRN2 | RPS18 | TMEM44 |
| SIRT5 | CTSL | PFKL | VDAC2 | RPS8 | CEP44 |
| ATP5F1C | RHOXF2 | ZNF391 | UTP11 | H2AC12 | MAIP1 |
| LZTS3 | TMEM263 | H1-10 | H2BC12 | OBI1 | RBM14-RBM4 |
| MIR137 | MIR148A |  |  |  |  |
